# Evaluation of Military Recruits With Complaints of Palpitations After Physical Training: A Study From Turkey

**DOI:** 10.7759/cureus.29284

**Published:** 2022-09-18

**Authors:** Serhat Günlü, Adem Aktan

**Affiliations:** 1 Cardiology, Dağkapı State Hospital, Diyarbakır, TUR; 2 Cardiology, Mardin Training and Research Hospital, Mardin, TUR

**Keywords:** cardiac conduction system, discharge, physical training, palpitations, military recruits

## Abstract

Objective

Recruits undergo medical examination before mandatory service. After enlistment, if recruits have health problems, they are sent to a medical board to establish fitness for their duties. We aimed to analyze the complaints of palpitations after physical training in recruits without a known history of cardiovascular disease (CVD) and determine whether the diagnoses were suitable for duty.

Methods

This cross-sectional descriptive study was conducted among 25,666 participants who were admitted to an outpatient cardiology clinic due to complaints of palpitations between August 2016 and June 2022. Information regarding socio-demographic characteristics was collected. Laboratory test results and electrocardiography (ECG) were analyzed. The diagnoses were evaluated.

Results

In total, 582 patients who were dismissed from the military were included in the study. The mean age of patients was 19.23±2.02 years. Among the patients, drug use (26; 6.2%) and history of addictive substance use (178; 30.6%) were low. The number of days of service under 10 was high (450; 77.3%). The prevalence of sleep disturbance (122; 21%) and hydration habits (154; 26.5%) were low. According to ECG findings, premature atrial contractions were high (250; 42.9%). There was a significant correlation between the ECG findings and seasons (p<0.001).Rheumatic valve disease (83; 14.26%) and supraventricular tachycardia (77; 13.23%) were the most common diagnoses.

Conclusion

2.2 percent of all participants admitted to the hospital due to palpitations were dismissed from the military service, and 0.7 percent of them were diagnosed with cardiac conduction system disease.

## Introduction

Military physical training is an integral element of personnel's daily lives and is essential for maintaining or enhancing physical performance. Failure to diagnose heart diseases in the medical examination performed before mandatory military service, on the other hand, may result in complaints of palpitations during physical effort [1].

Military duty is also inherently associated with harsh disciplinary processes, long working hours, unsuitable climatic and topographic circumstances, and fright of enemy action, all of which increase the risk of arrhythmias [2]. Symptomatic arrhythmias may limit the ability of military personnel to execute essential duties in various military occupations, impacting military readiness, deployment eligibility, and overall retention capacity. Therefore, those with illnesses are unfit for military services and should be dismissed [3].

In this study, we aimed to investigate the complaints of palpitations in Turkish military recruits and assess whether their diagnoses were suitable for duty.

## Materials and methods

Selection of participants

A cross-sectional descriptive study was conducted among 25,666 participants who were admitted to an outpatient cardiology clinic due to complaints of palpitations between August 2016 and June 2022. No evidence was found to explain the palpitations in 24,871 recruits. 589 patients with abnormal ECG findings were included in the study. Seven patients were omitted because of lack of data. Legal highs were defined as substances designed to produce effects similar to illicit drugs. The body mass index (BMI) was determined using weight and height measurements. Obesity was defined as a BMI of 30 kg/m2 or above. Data were collected from the archive of Dağkapı State Hospital, which has served as a military hospital since 1845. The data collection spreadsheet was kept separate from any personal identifying information, and all data were stored in a password-protected, encrypted file.

Study protocol

Routine blood tests were performed for all patients. ECG was performed using an electrocardiograph (model ECG-1350K Nihon-Kohden Corporation, Tokyo, Japan) at a rate of 25 mm/s and 10 mm/mV amplitude, which was reviewed by a cardiologist who was blinded to the study. A 24-hour standard 3-leads (leads V1, V2, and V5) Holter ECG (Northeast Monitoring, Maynard, USA) and echocardiography were performed (Philips ultrasonography Model HD7 XE, Philips Healthcare, Best, Netherlands) if necessary.

Ethical approval

The study protocol was authorized by the local ethics committee (Gazi Yaşargil Training and Research Hospital; no. 2022-110, June 24, 2022), and it followed the Declaration of Helsinki's ethical guidelines for human testing (2013).

Statistical methods

All analyses were performed using SPSS software version 24.0 (IBM Corp, Armonk, USA). The initial continuous variables were expressed as mean±standard deviation or median (interquartile range) according to the dispersion of the data. Frequency and percentage were used to express categorical variables. The Chi-squared test or Fisher’s exact test was used for categorical variables. The level of significance was set at p<0.05.

## Results

**Figure 1:**
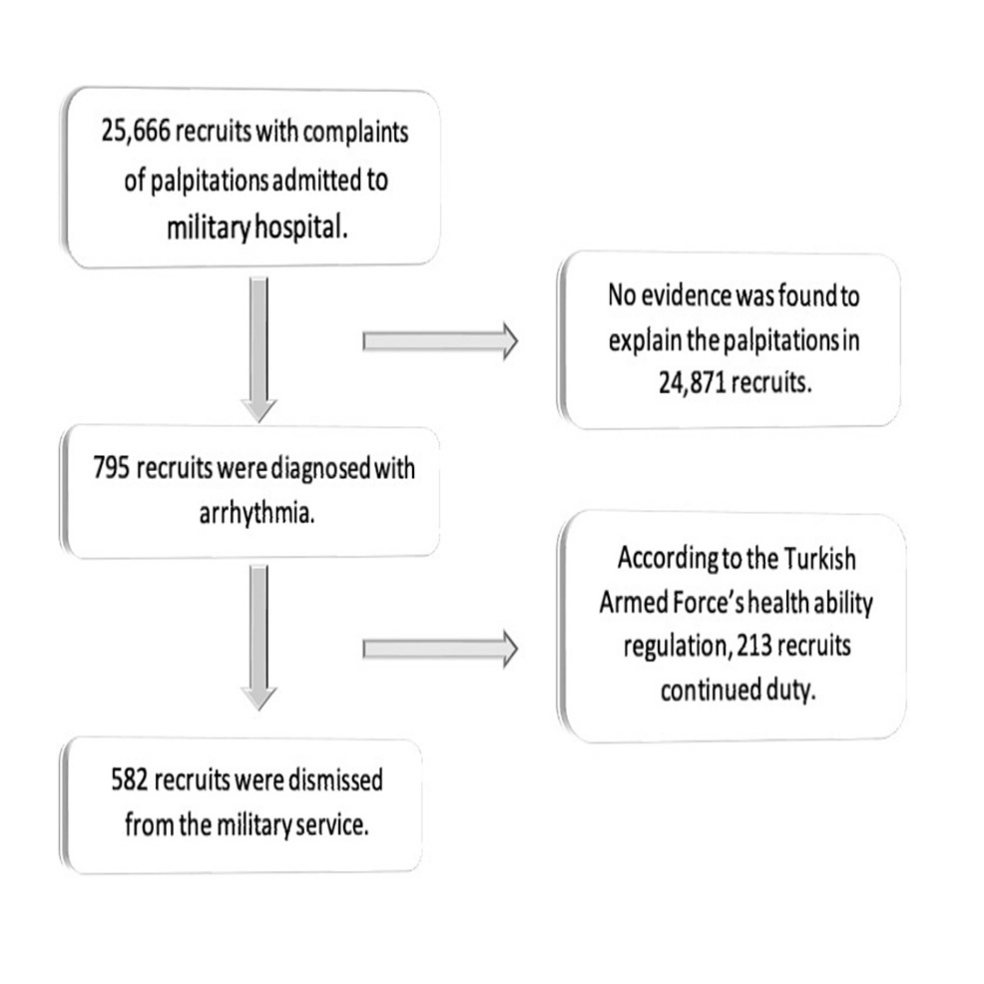
Flow diagram of the study

A total of 582 patients with a mean age of 19.23±2.02 years were included in the study. Figure 1 shows the flow diagram of the study.

Among the patients, 93.8% were not drug users, and 69.4% did not have a history of addictive substance use. 77.3% of recruits had been in the military for less than ten days of service. The prevalence of sleep disturbances (21%) and hydration habits (26.5%) was low. The average BMI (body mass index) values (68.9%) were in the normal range (Table 1).

**Table 1:** Socio-demographic characteristics and laboratory measurements of the study participants (n=582) Values are reported as mean ± SD, n (%), or median (interquartile range), and n (%) for categorical variables. Sodium (Na), Calcium (Ca), Iron (Fe), Potassium (K), Hemoglobin (HGB), Aspartate aminotransferase (AST), Alanine aminotransferase (ALT), White blood cell (WBC), Body mass index (BMI).

Parameters		x̄±SD, or[IQR]	Min-Max
Age (years)		19.23±2.02	18-38
Na (mmol/L)		139.4±3.43	128-147
Ca (mmol/L)		9.8±0.57	8.40-10.90
Troponin I (pg/mL)		19.72±4.72	5-32
D-dimer (ng/mL)		361 (288-496)	119-1765
Fe^+2^ (ug/dL)		93 (88-96)	26-112
K (mmol/L)		4.1 (3.7-4.4)	3.20-7.80
Hbg (g/dL)		14.1±1.4	9.30-17.11
Ast (U/L)		16 (12-25)	4-56
Alt (U/L)		21 (17-32)	10-97
Wbc (10^3^/mm^3^)		8.61 (7.2-10.5)	3.43-21.07
Characteristics		Number	Percentage
Hydration habit	No	428	73.5
	Yes	154	26.5
Sleep disturbance	No	460	79
	Yes	122	21
Days of service	<10 days	450	77.3
	10-30 days	120	20.6
	>30 days	12	2.1
BMI (kg/m^2^)	<18.9	112	19.2
	19-24.9	400	68.7
	>25	70	12.1
Drug use	None	546	93.8
	Anti-histamines	11	1.9
	Anti-psychotic	25	4.3
Addictive substance use history	None	404	69.4
	Alcohol	26	4.5
	Cigarette	114	19.6
	Legal highs	22	3.8
	Thinner	16	2.7

**Figure 2:**
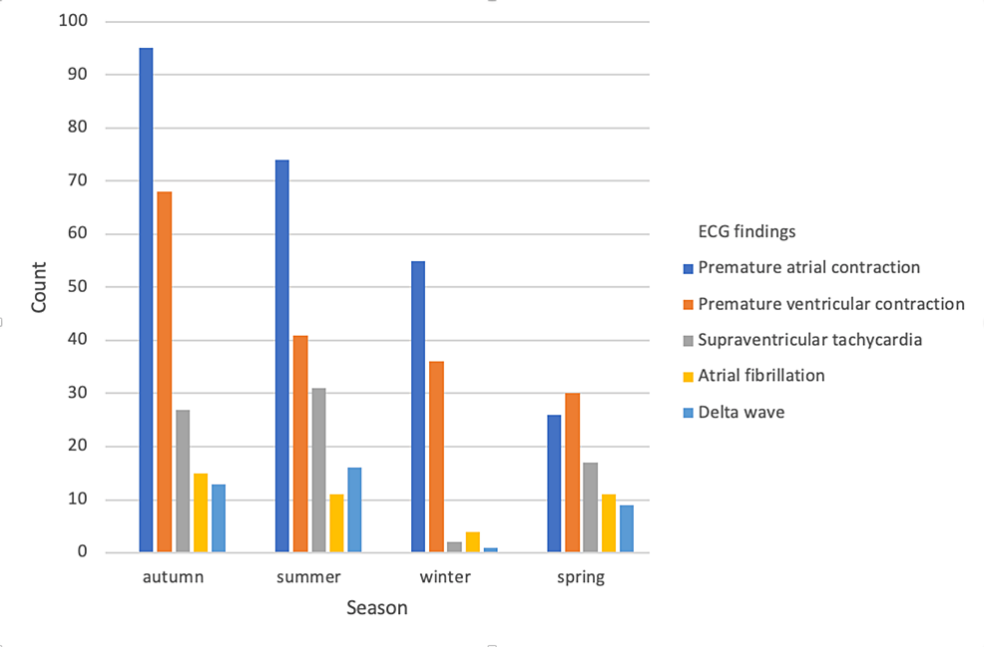
Seasonal variation of ECG findings

Premature atrial contractions (42.9%), premature ventricular contractions (30%), supraventricular tachycardia (13.2%), atrial fibrillation (7%), and delta waves (6.7%) were observed. There was a significant correlation between the ECG findings and seasons (p<0.01). The number of ECG findings according to the season is shown in Figure 2.

**Figure 3:**
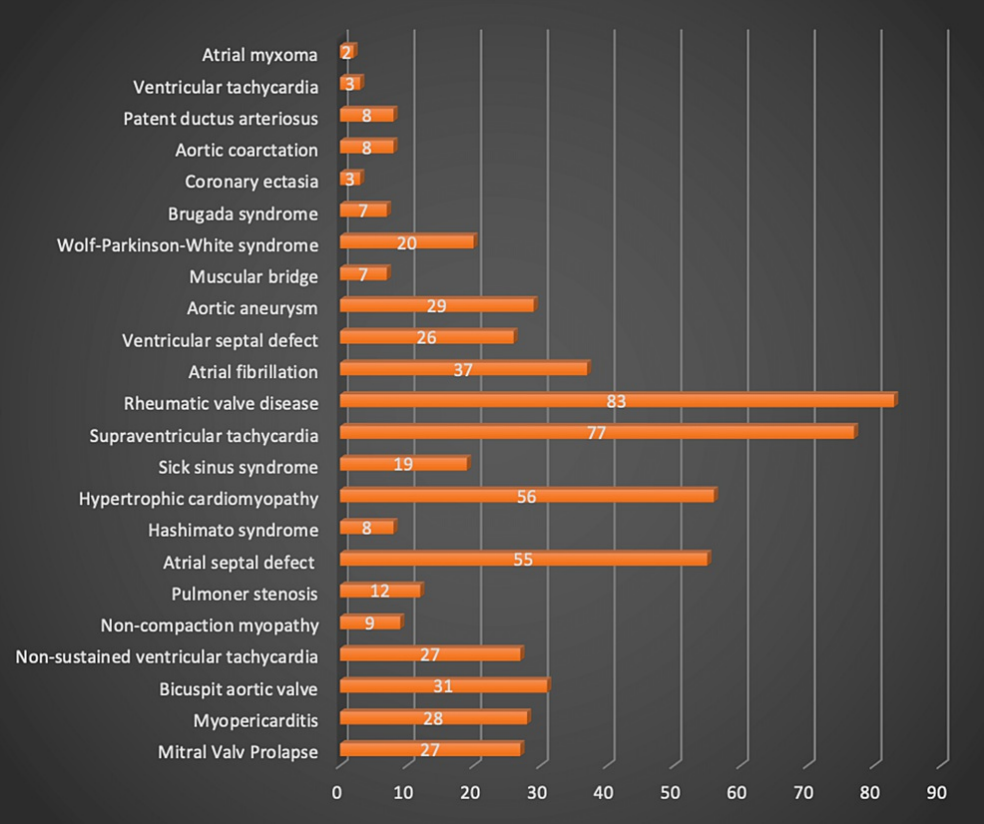
Frequency distribution of patients by diagnosis

Rheumatic valve disease (14.3%) and supraventricular tachycardia (13.2%) were detected as the most common diagnosis. The frequency distribution of patients by diagnosis is shown in Figure 3.

## Discussion

This study aimed to offer a profile of recruits complaining of palpitations after physical training in the Turkish army. Heart conduction system disease was found in 0.7% of all participants, and supraventricular tachycardia was the most common disease that was missed (0.3%) during the medical exam before mandatory military service.

CVD is less common in recruits than in the general population. The military population consists mainly of young people. Palpitation is common among recruits. It is usually due to electrophysiological, genetic, and congenital structural heart disease [2,3]. In addition, substance use, climate and environmental conditions, extreme physical training, fluid and electrolyte balance disorders, cardiac complications of febrile diseases, and overweight cause palpitation development.

Sleep disturbance, hydration habits, days of service for motivation, and climatic conditions were identified as physiological mechanisms of the palpitations [1,3]. The military environment creates challenges for sound sleep. Military personnel often underestimate the importance of sleep. Luxton et al. reported that recruits who slept for less than five hours were more injured during physical training and were more wounded during combat [4]. This situation endangers the safety of both the person and the unit. Thus, the probability of discharge from the military increases [5]. Carretero-Krug et al. reported that inadequate hydration during exercise increases anxiety and that palpitations negatively affect mental performance [6]. Heat strokes are common in the Middle East. Venuto et al. reported that sudden death from heat stroke was common in the US Middle East military unit [7]. However, they reported that palpitations in winter were caused by rheumatoid valve involvement and myocarditis. In a study conducted in Sweden, 63.2% of recruits hospitalized at 14 years of follow-up were diagnosed with myocarditis [8].

Alcohol and cigarette use are increasing daily among young people. It has been reported as a cause of syncope attacks during training in New Zealand recruits. It also increased health expenditures as it caused serious injuries [9]. Quednow et al. reported that 69% of the Switzerland population used alcohol and cigarettes [10]. Legal highs were used by 49% of youth over the age of 20, and more than 10% of legal highs were synthetic [11]. This may endanger the future post-service health of recruits. In the United States, access to substances is easier and more socially accepted. Legal highs use is more prevalent between the ages of 18 and 30 [12]. Medical officers across the nation should provide briefings to improve the health of military members and the combat readiness of the armed forces [13].

Arrhythmia can occur in a structurally normal heart. Hasija et al. diagnosed 15 recruits with supraventricular tachycardia between 2014 and 2016 at an Indian military hospital [14]. Brugada syndrome, which causes sudden cardiac death during exercise, is observed in 1% of young people [15]. This is rare in military recruits. Guettler et al. detected it in only one patient among a 300-man German aircrew [16]. Murphy et al. diagnosed Wolff-Parkinson-White syndrome (WPW) in seven and Brugada syndrome in one of the recruits [17]. In the American army, 386 paroxysmal atrial fibrillation (AF) emerged in 15 years of follow-up, 40% of which were deployed [18]. 

The side effects of medications and endocrine abnormalities can lead to arrhythmia. Anti-histaminic agents may cause sudden death owing to QT prolongation [19,20]. Another agent that prolongs the QT interval, which is also accepted by neuropsychiatrists, is anti-psychotics [21]. Metabolic syndromes can also cause arrhythmia. Metabolic syndrome was detected in 24.3% of the Saudi army population, and 578 of them had frequent premature atrial contractions [22]. In a study by Palle, 512 overweight recruits developed significant arrhythmias during exercise [23].

Arrhythmia can occasionally be seen as a symptom of cardiac abnormalities. McKenna and Judge planned to diagnose congenital structural heart disease using echocardiography during the pre-enlistment examination [24]. They found 1.3% of structural heart diseases in the study. Thus, the recruitment of young people with atrial and ventricular septal defects was prevented. Liu et al. diagnosed mitral valve prolapse (MVP) in 82 of 2442 recruits in the Taiwan army [25]. Patients with cardiomyopathy who exercise are at risk for sudden cardiac death. In these patients, ventricular tachycardia is induced by exercise. Sudden cardiac death (SCD) was not seen in our patients because most recruits had less than 10 days of military service.

Limitations

The Turkish government exempts women from mandatory military service. Owing to the gender disparity, this could spark some debate. Our sample does not cover the entire Turkish army.

## Conclusions

In our study, recruits who developed palpitations due to modifiable reasons continued their duties. Non-modifiable causes such as structural heart diseases, which may cause dismissal from the military were common among recruits who complained of palpitations. Previously undiagnosed patients may present symptoms during strenuous physical training. Routine pre-military echocardiographic evaluation and ECG stress test should be performed before enlistment into the military. Thus, we can reduce heart diseases which can cause palpitations in recruits.
